# Multisensory perception of the six basic emotions is modulated by attentional instruction and unattended modality

**DOI:** 10.3389/fnint.2015.00001

**Published:** 2015-02-02

**Authors:** Sachiko Takagi, Saori Hiramatsu, Ken-ichi Tabei, Akihiro Tanaka

**Affiliations:** ^1^Tokyo Woman’s Christian UniversityTokyo, Japan; ^2^Waseda Institute for Advanced StudyTokyo, Japan; ^3^Graduate School of Medicine, Mie UniversityTsu, Japan

**Keywords:** attentional instruction, audiovisual integration, unattended stimuli, modality dominance, congruency effect, emotion perception

## Abstract

Previous studies have shown that the perception of facial and vocal affective expressions interacts with each other. Facial expressions usually dominate vocal expressions when we perceive the emotions of face–voice stimuli. In most of these studies, participants were instructed to pay attention to the face or voice. Few studies compared the perceived emotions with and without specific instructions regarding the modality to which attention should be directed. Also, these studies used combinations of the face and voice which expresses two opposing emotions, which limits the generalizability of the findings. The purpose of this study is to examine whether the emotion perception is modulated by instructions to pay attention to the face or voice using the six basic emotions. Also we examine the modality dominance between the face and voice for each emotion category. Before the experiment, we recorded faces and voices which expresses the six basic emotions and orthogonally combined these faces and voices. Consequently, the emotional valence of visual and auditory information was either congruent or incongruent. In the experiment, there were unisensory and multisensory sessions. The multisensory session was divided into three blocks according to whether an instruction was given to pay attention to a given modality (face attention, voice attention, and no instruction). Participants judged whether the speaker expressed happiness, sadness, anger, fear, disgust, or surprise. Our results revealed that instructions to pay attention to one modality and congruency of the emotions between modalities modulated the modality dominance, and the modality dominance is differed for each emotion category. In particular, the modality dominance for anger changed according to each instruction. Analyses also revealed that the modality dominance suggested by the congruency effect can be explained in terms of the facilitation effect and the interference effect.

## INTRODUCTION

Human beings must perceive other people’s emotions appropriately to facilitate successful social interactions. Emotions are expressed using different sensory channels, such as a face and voice, and are judged by integrating information from these channels in natural settings. Previous studies have shown that the perceptions of facial and vocal affective expressions are interactive. For instance, emotional judgments based on one modality are impaired by incongruent emotions and enhanced by congruent emotions expressed in other modalities (de Gelder and Vroomen, 2000; Kreifelts et al., 2007; Collignon et al., 2008). These findings have been confirmed by experiments using both static faces (Massaro and Egan, 1996; de Gelder et al., 1999; de Gelder and Vroomen, 2000) and dynamic faces (Collignon et al., 2008; Van den stock et al., 2008; Tanaka et al., 2010). Furthermore, integration of emotional information from the face and voice has been demonstrated in infants (Grossmann et al., 2006) and people with pervasive developmental disorder (Magnèe et al., 2008). Results from brain studies have shown that emotions from the face and voice interact with each other (Pourtois et al., 2005; Ethofer et al., 2006a,b; Kreifelts et al., 2007; Talsma et al., 2007, 2010). Specifically, neuroimaging data using functional magnetic resonance imaging (fMRI) on audiovisual integration of emotional information highlights stronger activation in the left middle temporal gyrus (Pourtois et al., 2005), left basolateral amygdala (Ethofer et al., 2006a), right fusiform gyrus (Ethofer et al., 2006a), right thalamus (Kreifelts et al., 2007), and posterior superior temporal sulcus (Ethofer et al., 2006b; Kreifelts et al., 2007).

In most behavioral studies, participants were instructed to pay attention to only one modality (i.e., face or voice) and judge the emotion shown in that modality. This method allows us to investigate whether the emotional information from the face and voice are integrated inevitably. This paradigm, the immediate cross-modal paradigm (Bertelson and de Gelder, 2004), has been widely used to examine the multisensory perception of the emotion (de Gelder and Vroomen, 2000; Vroomen et al., 2001; Collignon et al., 2008). For instance, de Gelder and Vroomen (2000) asked participants to judge the emotion of congruent and incongruent face–voice stimuli expressing two opposing emotions (happiness and sadness) by instructing participants to attend to a certain modality. The results showed that accuracy of the emotion perception was higher for congruent stimuli than it was for incongruent stimuli. That is, though participants understood that they should pay attention to only one modality, the emotion perception was impaired by the emotion of the other modality in the presence of incongruent stimuli. Vroomen et al. (2001) examined whether integration of emotional information from the face and voice requires limited attentional resources using dual-task methodology. The results showed that emotional judgment from the face and voice was unconstrained by attentional resources. These findings imply that audiovisual integration of emotional information occurs as a mandatory process.

Is perceived emotion different according to whether participants are instructed to pay attention to either the face or voice or not? Few studies have compared the emotion perceptions with and without specific instructions regarding the modality to which attention should be directed. Collignon et al. (2008) conducted two experiments, one with instructions and one without instructions. They used stimuli denoting expressions of fear and disgust. Stimuli were faces and voices in which fear, disgust, or combinations of both were expressed. Experiment 1 without instructions used congruent multisensory stimuli and unisensory stimuli, and Experiment 2 with instructions used incongruent stimuli in addition to stimuli in Experiment 1. In both experiments, participants were asked to categorize fear and disgust. The results revealed that the performance on congruent multisensory stimuli was higher than that on unisensory stimuli in both experiments. That is, with regard to the emotion perception for unisensory and congruent multisensory stimuli, the results were consistent regardless of whether instructions as to attention were given. However, it is unclear whether the emotion perception for incongruent stimuli is modulated by attentional instructions.

Previous studies have also suggested that particular emotional channels dominate other channels when comparing the accuracy of emotion judgments in the unisensory condition against the multisensory condition. Collignon et al. (2008) suggest that performance of emotion judgment was better when participants attended to the face as opposed to the voice, at least for fear and disgust. Similarly, other research has reported higher accuracy for faces when compared to voices, even when only one of the two was presented (e.g., Johnstone and Scherer, 2000; Pell, 2002; Hawk et al., 2009).

Most studies investigating the audiovisual integration and modality dominance in judging emotions have evaluated a limited number of emotions (sometimes as few as two). However, recent studies have focused on more than two emotions and have shown face dominance in general. Paulmann and Pell (2011) used congruent face–voice pairs in which five emotions (anger, disgust, sadness, happiness, and surprise) and neutral were expressed. They investigated whether the emotion perception is more accurate for multi-channel stimuli by presenting stimuli with different combinations of the face and prosody of the voice. Participants were not given specific instructions with regard to attention. The emotion perception was better in response to multi-channel as opposed to single-channel stimuli. When stimuli contained only one emotional channel, perception tended to be higher for faces than for vocal prosody. However, this tendency was not uniform across emotion categories. Föcker et al. (2011) used both congruent and incongruent face–voice pairs in which three emotions (happiness, anger, and sadness) and neutral were expressed. They aimed to test whether participants were able to categorize the stimuli based on each emotion, expecting that the accuracy on incongruent stimuli would be lower than that on congruent stimuli. Participants were instructed to pay attention to one modality. The accuracy on congruent audiovisual stimuli was higher than that on unisensory stimuli, and the accuracy on unisensory stimuli was higher than that on incongruent audiovisual stimuli. However, the accuracies varied across emotion categories. That is, both studies showed that the emotion perception accuracy is not uniform across emotion categories. Therefore, it is necessary to closely examine the emotion perception for each emotion category.

As presented above, previous studies have revealed that emotional information from the face and voice demonstrated mandatory interaction, and that facial cues generally dominate vocal cues in judging emotions from the face and voice. However, it is unclear whether the interaction of emotional information from the face and voice is mandatory for any emotion categories. Also it remains unclear whether the modality dominance is the same across emotion categories. It is important to examine the emotion perception in terms of the impact of instructions and the unattended modality using the six basic emotions.

In the present study, we examined whether the emotion perception is modulated by instructions to pay attention to one of two modalities. We used faces and voices expressing the six basic emotions, and face–voice combinations in which the face and voice showed the same or different emotion.

## MATERIALS AND METHODS

### PARTICIPANTS

Twenty-six Japanese university students residing in Japan (13 male, 13 female; average age 20.3 ± SD 1.4) participated in the experiment. All participants provided written informed consent prior to participation. The study was approved by the local ethics committee and all subjects gave their written informed consent prior to inclusion in the study.

### STIMULI SELECTION

#### Models

Twenty-one Japanese (10 male, 11 female) students demonstrated the six basic emotions for audiovisual speech stimuli.

#### Creation of the audiovisual speech stimuli

For the models’ utterances, six short phrases with emotionally neutral meanings were chosen. The models were asked to say “*Soonandesuka?*” (Is that so?), “*Korenani?*” (What’s this?), “*Sayoonara*” (Goodbye), “*Hai, moshimoshi*” (Hello), “*Doonatteruno?*” (What’s going on?), and “*Daijoobu?*” (Are you okay?) in Japanese with angry, disgusted, fearful, happy, sad, and surprised expressions. While facially expressing each intended emotion, the models uttered the six meaning-neutral phrases, filling them with the required emotion. Before starting the recording of each emotion, each model was instructed on how to facially and vocally perform the emotional expression. For facial expressions, instructions were given based on the Action Units of Ekman and Friesen (1978). For vocal expressions, samples by radio announcers were given and, when necessary, emotional context was provided to induce each emotion. After receiving these instructions, the models used a mirror to practice their expressions. The recording began when the model could adequately convey the emotion with simultaneous facial and vocal expressions. For the recording, they were asked to speak the phrases at three different speech rates—slow, normal, and fast—and to repeat them three times at each speech rate. Thus, 324 samples (6 emotions × 6 utterances × 3 speech rates × 3 repetitions) were recorded from each model.

A recording studio was used with sufficient lighting equipment on the ceiling. A digital video camera (SONY PVW-637 k) was used for recording video and a microphone (SONY ECM-77B) was used for recording audio. A gray background was used throughout the recording. The recordings took place per emotion type. All models wore a white cardigan and a pin microphone about 15 cm away from their mouths on their chests for recording audio. They sat about 2 m away from the camera and 30 cm in front of the background.

The recorded video was edited using Avid Xpress (Avid Technology, Inc.). For each model’s performance, the onset and offset of the utterance was identified. Then, we extracted the clip including five frames before and five frames after the onset. From the 21 models, eight models were selected via agreement between two evaluators who judged that the facial and vocal expressions suitably expressed each emotion. The selection standards consisted mainly of whether the differences among emotions were clearly discriminated in facial expressions and whether there were minimal head movements and blinking. Regarding vocal expressions, models were selected according to whether differences among emotions were clearly expressed and whether the utterance was fluent and clear. Furthermore, for each combination of the emotion and utterance, a total of nine video clips were recorded from each model, having repeated each utterance three times at three speech rates. From the nine video clips recorded, three were selected that had approximately the same utterance duration, regardless of the instruction on speech rate. Two uttered phrases were eliminated. One phrase (“*Daijoobu?*”) was eliminated because some participants pointed out that it does not have a neutral meaning. The other phrase (“*Hai, moshimoshi*”) was eliminated because it has a pause between “*Hai*” and “*moshimoshi*” that makes it difficult to create incongruent stimuli. Finally, the uttered phrases were reduced to just four: “*Soonandesuka?*” (Is that so?), “*Korenani?*” (What’s this?), “*Sayoonara*” (Goodbye), and “*Doonatteruno?*” (What’s going on?).

#### Evaluation experiment

For the evaluation experiment, participants were 99 Japanese university students (47 male, 52 female; average age 20.7 ± SD 2.07). The experiment was divided into two rounds, with only facial expressions in the first round and only vocal expressions in the second round. The reason for keeping the order of rounds constant was that it was likely to preclude biased evaluations of the facial expressions due to lip-reading from the uttered phrase already being known. Both rounds were conducted in groups (10–20 participants), and participants were required to participate in both rounds. The experiment consisted of a total of eight sessions, corresponding to each of the eight models, for a total of 72 trials. The order of the sessions was counterbalanced. Images were projected onto a screen in the front of the classroom using a projector attached to a PC, and sound was presented through a loudspeaker. Participants were seated in a spot from which they could adequately see the entire screen. They were instructed to choose which the emotion was being expressed (or heard) from the six emotions (anger, disgust, fear, happiness, sadness, and surprise) and write on an answer form that was provided. Further, they were instructed to judge intuitively rather than think deeply about their decision.

The rates of matches between participants’ responses and models’ intended emotions were calculated per model and uttered phrase. Based on the results, the four models and two uttered phrases, “*Soonandesuka?*” (Is that so?) and “*Doonatteruno?*” (What’s going on?), that generated the most matches were used in the main experiment. The mean accuracies of emotion judgment from faces and voices for selected stimuli with respect to the emotion category are shown in **Table 1**.

**Table 1:** Mean accuracies (%) of emotion judgment from faces and voices for selected stimuli with respect to the emotion category (SD in parentheses).

	Emotion category					
	Anger	Disgust	Fear	Happiness	Sadness	Surprise
Face	80.3	73.0	21.3	98.6	69.7	83.7
	(15.05)	(9.88)	(6.46)	(1.98)	(32.12)	(8.53)
Voice	64.4	49.3	31.9	62.4	62.4	73.6
	(24.28)	(9.79)	(20.22)	(28.98)	(33.33)	(13.80)

Based on the above result, faces and voices presented in the evaluation experiment were edited and processed so that emotions depicted by the facial expressions and vocal expressions were paired in a congruent or incongruent fashion. The former were congruent stimuli, and the latter were incongruent stimuli of the main experiment. There were 36 combinations of facial expression (6) and vocal expression (6) in total. In these combinations, six combinations were congruent and 30 combinations (6 × 5) were incongruent. Finally, there were 48 congruent stimuli (6 congruent combinations × 2 phrases × 4 actors) and 240 incongruent stimuli (30 incongruent combinations × 2 phrases × 4 actors).

### PROCEDURE

The main experiment was conducted in a group setting. Visual and audio stimuli were presented in the same way as the evaluation experiment. Participants were seated such that they could see the entire screen and listen to the auditory stimuli. In the main experiment, there were unisensory and multisensory sessions. In the unisensory session, only faces or voices were presented. The multisensory session was divided into three blocks according to whether an instruction was given to pay attention to a given modality. In the no instruction (NI) block, participants were not instructed to pay attention to either of the two modalities. In the visual instruction (VI) block, participants were instructed to pay attention to the visual information (i.e., face). In the auditory instruction (AI) block, participants were instructed to pay attention to the audio information (i.e., voice). Participants were required to judge the emotion perceived from both the face and voice, the face only, and the voice only in NI, VI, and AI, respectively. They were required to ignore the voice in VI and the face in AI. The main experiment always began with the multisensory session. In the multisensory session, the first block was NI, followed by VI and AI. The order of VI and AI was counterbalanced. In the unisensory session, the order of the face and voice blocks was also counterbalanced. Participants answered the judged the emotion in handwriting by choosing one of the six options on the answer sheet.

Each block of the multisensory session consisted of 288 trials and each block of the unisensory session consisted of 192 trials (96 stimuli repeated two times). Stimuli were presented in random order in all blocks of both sessions. The main experiment took 2 h per day for 2 days. Participants were allowed to take a 10 min break every hour.

## RESULTS

In this study, we examined whether the emotion perception is modulated by instructions to pay attention to one of two modalities. We used both congruent and incongruent stimuli expressing the six basic emotions. The modality dominance between the face and voice for each emotion was also examined. We describe the results according to these purposes in the following sections.

### EMOTION PERCEPTION WITH AND WITHOUT INSTRUCTIONS ON INCONGRUENT TRIALS

Here, we focused on only incongruent trials in NI, VI, and AI. To examine the emotion perception when NI to pay attention to one modality were given, we performed a response modality (face or voice responses) × emotion (anger, disgust, fear, happiness, sadness, or surprise) two-way analysis of variance (ANOVA) on participants’ responses on incongruent trials in NI. A significance level of *p* < 0.05 was used for all ANOVA to evaluate all effects. Face responses for each emotion were defined as the mean percentage of participants’ responses for a given emotion when the face expressed that emotion. For example, when presented with stimuli in which the face showed anger but the voice showed a different emotion, we calculated the mean percentage of “anger” responses as the face response for anger. In contrast, voice responses for each emotion were defined as the mean percentage of responses for a given emotion when the voice expressed that emotion. For example, when presented with stimuli in which the voice expressed anger but the face expressed a different emotion, we calculated the mean percentage of “anger” responses as the voice response for anger. We used the face and voice responses for each emotion as the dependent variables.

**FIGURE 1:**
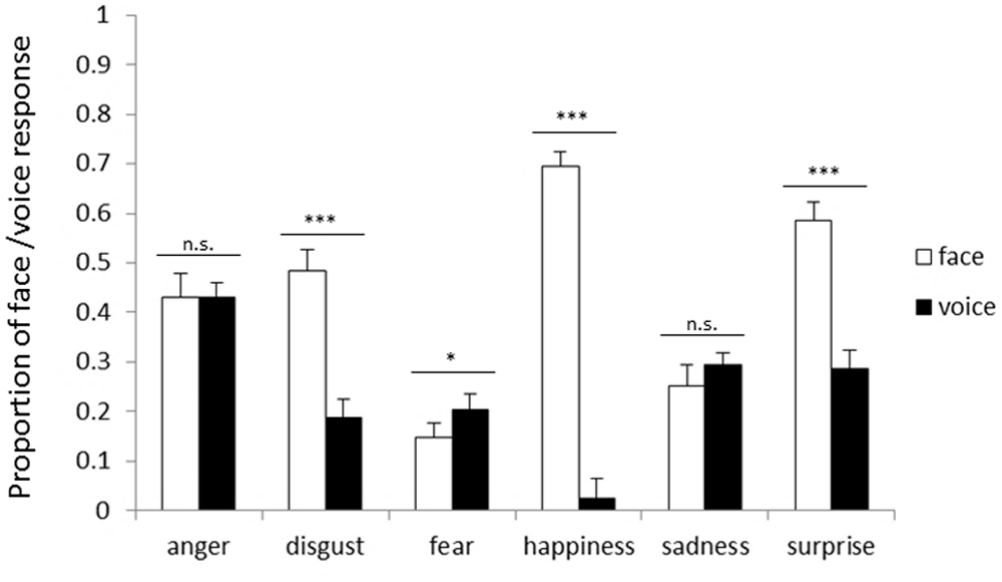
**Proportion of facial and vocal response on incongruent trials for each emotion category in the no instruction (NI) block.** Error bars represent SE. Asterisks indicate significant differences between modalities (*p < 0.05, ***p < 0.001).

**Figure 1** shows the proportion of the face and voice responses for each emotion in NI. The main effect of response modality was significant [*F*(1,25) = 106.86, *p* < 0.001]. The proportion of the face responses (43.2%) was higher than that of voice responses (23.8%, *p* < 0.001), demonstrating that facial cues dominated vocal cues when MI was given to pay attention to one modality. The main effect of emotion was also significant [*F*(5,125) = 21.57, *p* < 0.001], and the proportion for fear was the lowest (17.6%, *p*s < 0.05). In addition, the two-way interaction between response modality and emotion was significant [*F*(5,125) = 156.50, *p* < 0.001]. Simple main effects analyses showed that the proportion of the face responses was higher than that of the voice responses for disgust (face 48.4%, voice 18.7%), happiness (face 69.4%, voice 2.4%), and surprise (face 58.6%, voice 28.7%; *p*s < 0.001). In contrast, the proportion of the voice responses (20.4%) was higher than that of the face responses for fear (14.8%, *p* < 0.05).

To examine the emotion perception when the instruction to pay attention to one modality was given, a similar analysis was applied to VI and AI. We performed an attended modality (VI or AI) × emotion (anger, disgust, fear, happiness, sadness, or surprise) two-way ANOVA on the accuracy on incongruent trials in VI and AI. The accuracy for each emotion was calculated according to the attended modality. For example, when presented with stimuli in which the face showed anger but the voice showed a different emotion in VI, we calculated the proportion of “anger” responses as the accuracy for anger in VI. In contrast, for stimuli in which the voice showed anger but the face showed a different emotion in AI, we calculated the proportion of “anger” responses as the accuracy for anger in AI. We used these accuracies for each emotion as the dependent variables.

**FIGURE 2:**
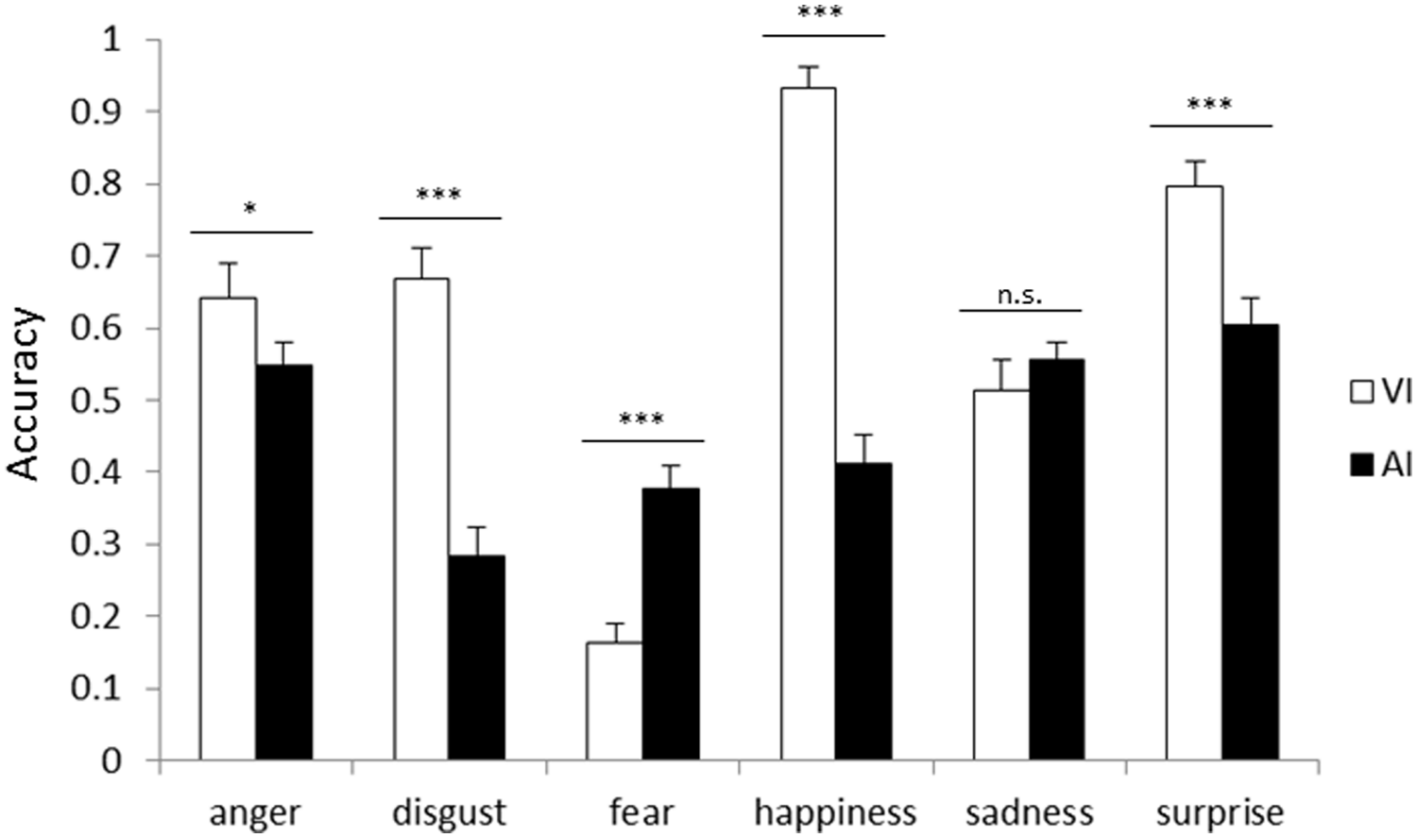
**Accuracy for each emotion category in visual instruction (VI) block and auditory instruction (AI) block.** Error bars represent SE. Asterisks indicate significant differences between modalities (*p < 0.05, ***p < 0.001).

**Figure 2** shows the accuracies for each emotion in VI and AI. The main effect of the attended modality was significant [*F*(1,25) = 48.16, *p* < 0.001]. The accuracy in VI (61.9%) was higher than that in AI (46.3%, *p* < 0.001). This showed that facial cues dominated vocal cues when the instruction to pay attention to one modality was given. The main effect of emotion was also significant [*F*(5,125) = 37.76, *p* < 0.001], and the accuracy for fear was the lowest (27.1%, *p*s < 0.05). In addition, the two-way interaction between the attended modality and emotion was significant [*F*(5,125) = 44.71, *p* < 0.001]. Simple main effects analyses showed that the accuracy in VI was higher than that in AI for anger (VI 64.2%, AI 54.9%; *p* < 0.05), disgust (VI 66.7%, AI 28.4%), happiness (VI 93.3%, AI 41.2%), and surprise (VI 79.6%, AI 60.5%) (*p*s < 0.001). In contrast, the accuracy for fear was higher in AI (37.8%) than it was in VI (16.3%; *p*s < 0.001).

The comparison between 1 and 2 based on the analyses showed that facial cues generally dominate vocal cues, regardless of the presence of instructions. For anger, face dominance was shown only when paying attention to one modality.

### IMPACT OF CONGRUENCY OF EMOTIONS BETWEEN FACES AND VOICES

In the previous section, we focused on only incongruent trials in NI, VI, and AI. Here, to examine the emotion perception in terms of congruency between facial and vocal cues, we focused on both congruent and incongruent trials when instructions to pay attention to one modality were given. We also investigated whether the channel being unisensory or multisensory had an effect. We performed an attended modality (face or voice) × presentation condition [multisensory_congruent (MC), unisensory (UNI), or multisensory_incongruent (MI)] × emotion (anger, disgust, fear, happiness, sadness, or surprise) three-way ANOVA on the accuracy in each presentation condition.

**FIGURE 3:**
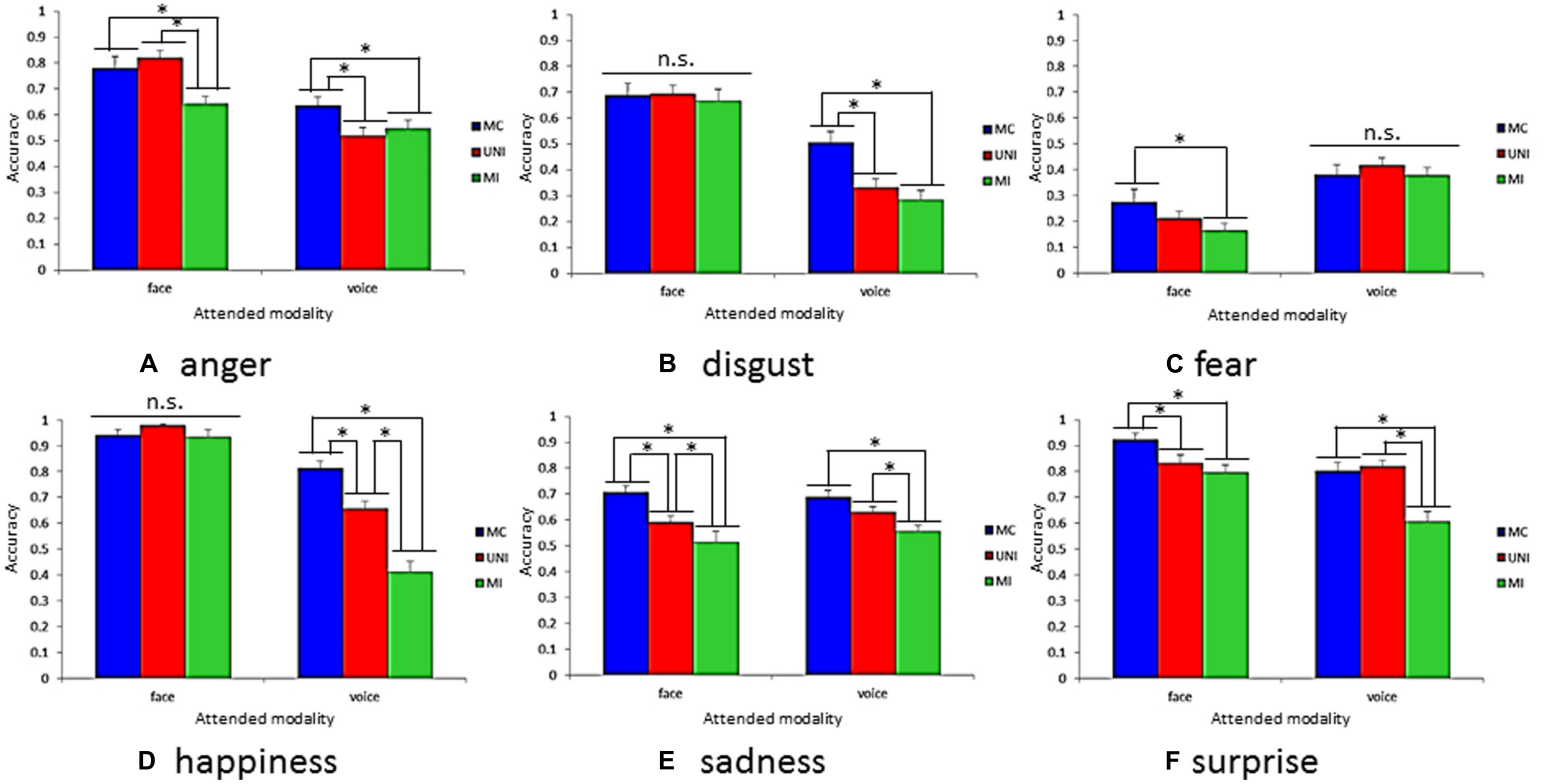
**Accuracy on all presentation conditions for each emotion category with respect to attended modality. (A)** anger, **(B)** disgust, **(C)** fear, **(D)** happiness, **(E)** sadness, and **(F)** surprise. Error bars represent SE. Asterisk indicate significant differences between presentation conditions (*p < 0.05).

**Figure 3** shows accuracies on all presentation conditions for each emotion category with respect to each modality. Results revealed a significant three-way interaction [*F*(10,250) = 15.93, *p* < 0.001]. There were significant simple interactions between the attended modalities and presentation conditions for anger [*F*(2,300) = 12.67, *p* < 0.001], disgust [*F*(2,300) = 13.23, *p* < 0.001], fear [*F*(2,300) = 0.02, *p* < 0.05], happiness [*F*(2,300) = 41.33, *p* < 0.001], and surprise [*F*(2,300) = 8.86, *p* < 0.001]. The subsequent analysis revealed simple–simple main effects of the presentation conditions for each attended modality and emotion categories. The simple–simple main effect of the presentation conditions was significant for anger [*F*(2,600) = 17.06, *p* < 0.001], fear [*F*(2,600) = 6.15, *p* < 0.001], sadness [*F*(2,600) = 18.64, *p* < 0.001], and surprise [*F*(2,600) = 8.43, *p* < 0.001] when the attended modality was the face. Also, the simple–simple main effect of the presentation conditions was significant for anger [*F*(2,600) = 7.07, *p* < 0.001], disgust [*F*(2,600) = 26.65, *p* < 0.001], happiness [*F*(2,600) = 80.13, *p* < 0.001], sadness [*F*(2,600) = 8.71, *p* < 0.001], and surprise [*F*(2,600) = 28.18, *p* < 0.001] when the attended modality was the voice. In the next sections, we describe the results of multiple comparisons for a simple–simple main effect of the presentation conditions for each attended modality and emotion category.

**FIGURE 4:**
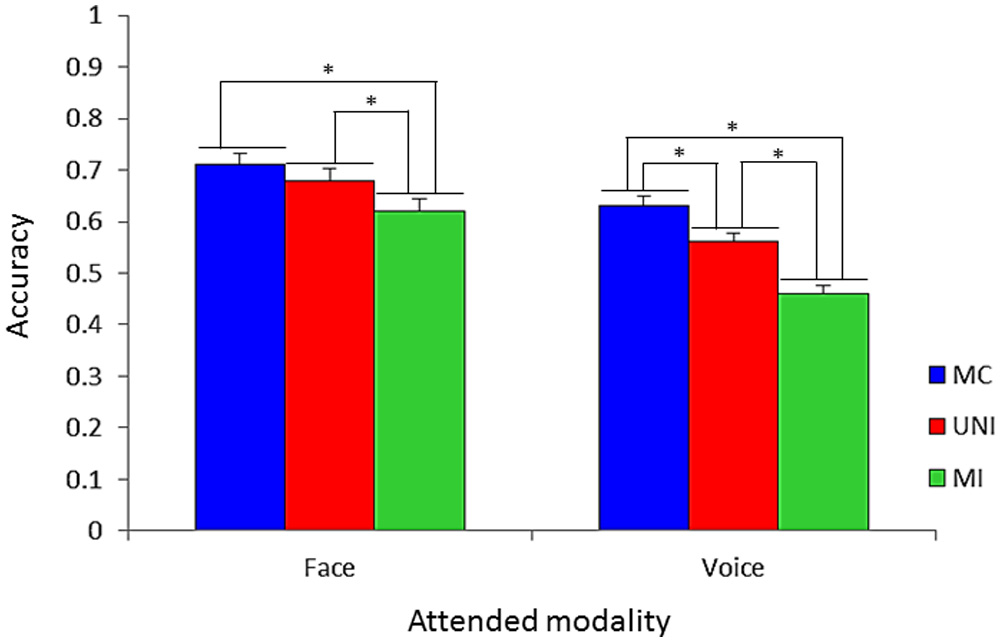
**Accuracy on all presentation conditions with respect to attended modality for all emotion categories.** Error bars represent SE (*p < 0.05).

Besides three-way interaction, it should be noted that an attended modality × presentation condition two-way interaction was also significant [*F*(2,50) = 6.13, *p* < 0.005]. That is, the accuracies among presentation conditions were different by modalities. **Figure 4** shows accuracies on all presentation conditions for all emotion categories included with respect to modality. The difference in the accuracies between MC (71.9%) and UNI (68.7%) was not significant, while the accuracies in MC and UNI were higher than that in MI (61.9%, *p*s < 0.05) when the attended modality was the face. In contrast, the accuracy in MC (63.7%) was higher than that in UNI (56.2%, *p* < 0.05), and the accuracy in UNI was higher than that in MI (46.4%, *p* < 0.05) when the attended modality was the voice.

#### Impact of presentation conditions

To examine the impact of presentation condition on the accuracy of the emotion perception for each attended modality and emotion, we describe the results of the multiple comparisons for simple–simple main effects of three-way interaction. For this purpose, we define the *congruency effect* as the difference in the accuracies between MC and MI. The congruency effect included two dissociable effects. Then, we defined the *facilitation effect* as the difference in the accuracies between MC and UNI and the *interference effect* as the difference in the accuracies between UNI and MI. Congruency effects, facilitation effects, and interference effects for each emotion category are shown as the results of multiple comparisons for the simple–simple main effects of presentation conditions for each attended modality and emotion (**Table 2**).

**Table 2:** Congruency, facilitation, and interference effects for each emotion category with respect to the attended modality.

	Face			Voice		
	Congruency effect	Facilitation effect	Interference effect	Congruency effect	Facilitation effect	Interference effect
Anger	○	–	○	○	○	–
Disgust	–	–	–	○	○	–
Fear	○	–	–	–	–	–
Happiness	–	–	–	○	○	○
Sadness	○	○	○	○	–	○
Surprise	○	○	–	○	–	○

*○, means significant, and –, means non significant in multiple comparisons for simple–simple main effect (*p* < 0.05).*

Congruency effects are represented by the difference in the accuracies between the blue and green columns in **Figure 3**. Multiple comparisons for simple–simple main effects of presentation conditions revealed that there were congruency effects for anger, fear, sadness, and surprise when the attended modality was the face. The accuracies in MC were higher than those in MI for these emotions (*p*s < 0.05). Furthermore, congruency effects were observed for all emotions except for fear when the attended modality was the voice. The accuracies in MC were higher than those in MI (*p*s < 0.05).

Facilitation effects are represented by the difference in the accuracies between blue and red columns in **Figure 3**. Multiple comparisons for simple–simple main effects of presentation conditions revealed facilitation effects for sadness and surprise when the attended modality was the face. The accuracies in MC were higher than those in UNI for these emotions (*p*s < 0.05). Furthermore, facilitation effects were shown for anger, disgust, and happiness when the attended modality was the voice. The accuracies in MC were higher than those in UNI (*p*s < 0.05).

Interference effects are reflected in the difference in the accuracies between the red and green columns in **Figure 3**. Multiple comparisons for simple–simple main effects of presentation conditions revealed that there were interference effects for anger and sadness when the attended modality was the face. The accuracies in UNI were higher than those in MI for these emotions (*p*s < 0.05). Furthermore, interference effects were shown for happiness, sadness, and surprise when the attended modality was the voice. The accuracies in UNI were higher than those in MI (*p*s < 0.05).

#### Modality dominance in congruency effect, facilitation effect, and interference effect

We examined the modality dominance for each emotion category based on the congruency effect, facilitation effect, and interference effect. Specifically, we performed an attended modality (face or voice) × emotion (anger, disgust, fear, happiness, sadness, or surprise) two-way ANOVA for the congruency, facilitation, and interference effects. It was assumed that the modality in which each effect was smaller dominated the other modality.

**FIGURE 5:**
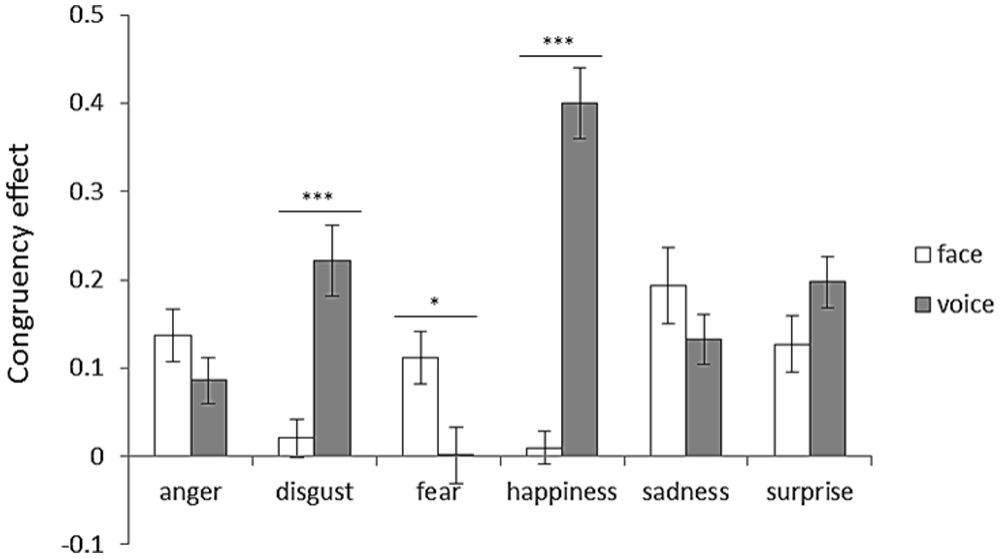
**Congruency effect for each emotion category with respect to attended modality.** Error bars represent SE. Asterisks indicate significant differences between attended modalities (*p < 0.05, ***p < 0.001). “V > A” in **Table 3** was the case that the bar length of the face was shorter than that of the voice, and “A > V” in **Table 3** was the case that the bar length of the voice was shorter than that of the face.

To examine the congruency effect, an attended modality (face or voice) × emotion (anger, disgust, fear, happiness, sadness, or surprise) two-way ANOVA was performed. **Figure 5** shows congruency effects for each emotion category with respect to the attended modality. Two-way ANOVA revealed significant interaction between the attended modality and emotion [*F*(5,125) = 21.91, *p* < 0.001]. The simple main effects revealed face dominance for disgust and happiness (*p*s < 0.001). In contrast, voice dominance was shown for fear (*p* < 0.05).

**Table 3:** Modality dominance as shown by congruency, facilitation, and interference effects for each emotion category.

	Congruency effect	Facilitation effect	Interference effect
Anger	–	V > A	A > V
Disgust	V > A	V > A	–
Fear	A > V	A > V	–
Happiness	V > A	V > A	V > A
Sadness	–	–	–
Surprise	–	A > V	V > A

*V > A means face dominance. A > V means voice dominance.*

**FIGURE 6:**
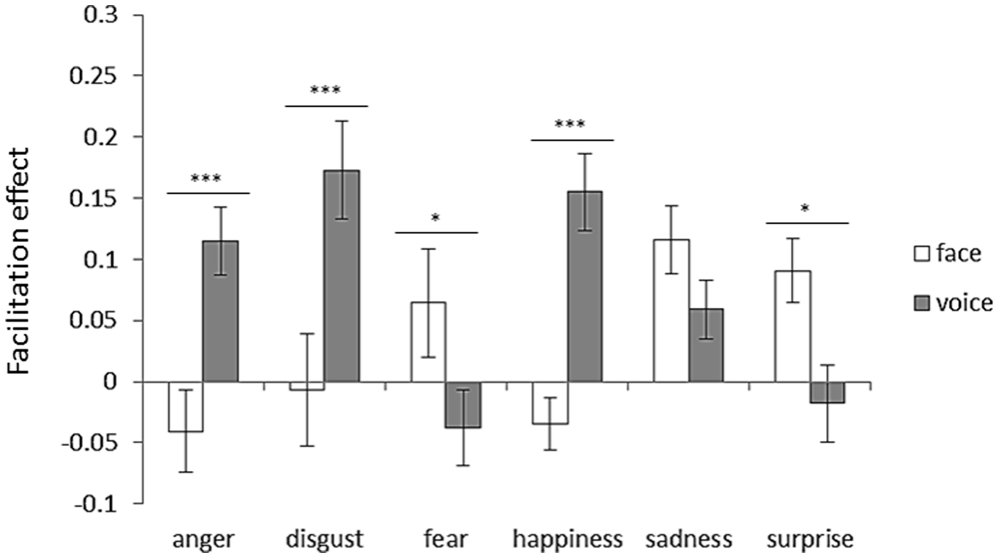
**Facilitation effect for each emotion category with respect to attended modality.** Error bars represent SE. Asterisks indicate significant differences between attended modalities (*p < 0.05, ***p < 0.001). “V > A” in **Table 3** was the case that the bar length of the face was shorter than that of the voice, and “A > V” in **Table 3** was the case that the bar length of the voice was shorter than that of the face.

To examine the facilitation effect, an attended modality (face or voice) × emotion (anger, disgust, fear, happiness, sadness, or surprise) two-way ANOVA was performed. **Figure 6** shows facilitation effects for each emotion category with respect to the attended modality. Two-way ANOVA revealed significant interaction between the attended modality and emotion [*F*(5,125) = 10.07, *p* < 0.001]. The simple main effects revealed face dominance for anger, disgust, and happiness (*p*s < 0.001). In contrast, voice dominance was shown for fear and surprise (*p*s < 0.05).

**FIGURE 7:**
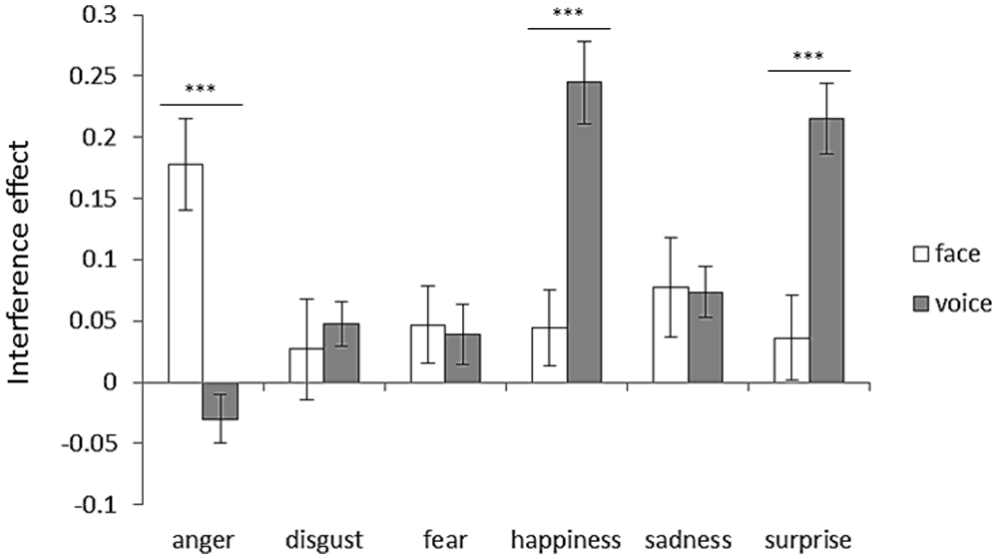
**Interference effect for each emotion category with respect to attended modality.** Error bars represent SE. Asterisks indicate significant differences between attended modalities (***p < 0.001). “V > A” in **Table 3** was the case that the bar length of the face was shorter than that of the voice, and “A > V” in **Table 3** was the case that the bar length of the voice was shorter than that of the face.

To examine the interference effect, an attended modality (face or voice) × emotion (anger, disgust, fear, happiness, sadness, or surprise) two-way ANOVA was performed. **Figure 7** shows interference effects for each emotion category with respect to the attended modality. Two-way ANOVA revealed significant interaction between the attended modality and emotion [*F*(5,125) = 17.88, *p* < 0.001]. The simple main effects revealed face dominance for happiness and surprise (*p*s < 0.001). In contrast, voice dominance was shown for anger (*p* < 0.001).

The modality dominances as shown by each effect for each emotion category are summarized in **Table 3**.

## DISCUSSION

The present study examined the impact of attention on the emotion perception of facial and vocal stimuli. Our results revealed that instructions to pay attention to one modality and congruency of emotions between modalities modulated the modality dominance, and the modality dominance differed by each emotion category.

### THE IMPACT OF ATTENTION ON EMOTION PERCEPTION OF FACES AND VOICES

Our results revealed face dominance in the audiovisual emotion perception. For most emotion categories, participants perceived the emotion from the face rather than from the voice, regardless of the modality to which participants were instructed to attend. This finding is in line with previous studies (Johnstone and Scherer, 2000; Pell, 2002; Collignon et al., 2008; Hawk et al., 2009). The emotion perception from faces is generally easier than from voices (Ekman and Friesen, 1971; Ekman et al., 1982; Russell et al., 1989; Russell, 1994; Scherer, 2003). In the previous studies examining the emotion perception from audiovisual stimuli when the attended instruction was given, the accuracies of emotion judgment from faces were generally higher than that from voices (Collignon et al., 2008; Föcker et al., 2011; Paulmann and Pell, 2011). Stimuli used in the current study showed similar tendency as shown in **Table 1**. Therefore, face dominance as shown in our results might have reflected this tendency.

More importantly, however, our results showed that the attentional instruction modulated the modality dominance for each emotion category. When the participants did not pay attention to any one modality, face dominance was shown for disgust, happiness, and surprise, while voice dominance was shown for fear. The modality dominance was not observed for anger or sadness. On the other hand, when participants paid attention to one modality, face dominance was shown for anger, disgust, happiness, and surprise, while voice dominance was shown for fear. The modality dominance was not observed for sadness. Therefore, the modality dominance for anger was modulated by the instruction. These results suggest that modality dominance was not consistent across emotion categories and that the modality dominance for anger was modulated by the instruction.

We speculate that this finding may be linked to the fact that the emotionally negative and threat information, especially anger and fear, is likely to capture the attention (Hansen and Hansen, 1988; Logan and Goetsch, 1993; Koster et al., 2004; Phelps et al., 2006; Bishop, 2008). In our study, face dominance was shown for anger when participants paid attention to one modality. This may simply reflect the fact that the emotion perception from faces is generally easier than that from voices (see **Table 1**). However, the modality dominance was not observed for anger when NI was given even though the same stimuli were presented. These findings might suggest that when NI was given, attention was automatically paid to one modality in which the threat information was expressed irrespective of whether it is the face or voice.

### THE IMPACT OF FACE–VOICE CONGRUENCY ON EMOTION PERCEPTION

The accuracy in congruent trials was higher than it was in incongruent trials. These results are in line with previous studies showing that the emotion perception improves when more than one source of congruent information about the intended emotion is available (Massaro and Egan, 1996; de Gelder and Vroomen, 2000; Pell, 2002; Collignon et al., 2008). In order to directly examine the modality dominance for each emotion category in terms of the congruency effect, the size of congruency effects between modalities was compared. The results showed face dominance for disgust and happiness, and voice dominance for fear (**Figure 5**; **Table 3**). The modality dominance was not observed for anger, sadness, or surprise (**Figure 5**; **Table 3**).

The congruency effect includes two opposing effects. One is the facilitation effect, which occurred when the same emotion as the attended modality was expressed in the unattended modality. The other is the interference effect, which occurred when a different emotion from that presented in the attended modality was expressed in the unattended modality. By comparing the accuracy in the unisensory condition with the accuracy in multisensory congruent and incongruent conditions, we were able to examine more precisely the modality dominance suggested by the congruency effect in terms of the facilitation and interference effects.

Regarding the facilitation effect, with all emotions included, the facilitation effect occurred only for the voice (**Figure 4**). Thus, if the emotions in the attended and unattended modalities were congruent, the vocal emotion perception was enhanced by the emotion shown in the face, while the emotion perception of faces was not enhanced by emotions portrayed in voices. Again, these findings demonstrate that facial cues generally dominate vocal cues in the emotion perception. For each emotion category, face dominance was present for anger, disgust, and happiness, whereas voice dominance was present for fear and surprise (**Figure 6**; **Table 3**). No modality dominance was observed for sadness (**Figure 6**; **Table 3**).

Among all included emotions, the interference effect occurred both for facial and vocal cues (**Figure 4**). Thus, if the emotion expressed in the unattended modality was different from that in the attended modality, the emotion shown in the unattended modality interfered with the emotion perception. These findings confirmed that emotional information from the face and voice are subject to mandatory integration (Massaro and Egan, 1996; de Gelder et al., 1999; de Gelder and Vroomen, 2000; Kreifelts et al., 2007). For each emotion category, the results showed face dominance for happiness and surprise, and voice dominance for anger (**Figure 7**; **Table 3**). The modality dominance was not observed for disgust, fear, or sadness (**Figure 7**; **Table 3**).

As mentioned above, the modality dominances present in the congruency effect, facilitation effect, and interference effect did not coincide. The relations between these modality dominances will be discussed in the next section.

### FACILITATION AND INTERFERENCE EFFECTS

As shown in the previous section and in **Table 2**, the modality dominance for each emotion was modulated not only by the instruction but also by congruency of emotions between modalities. Some of the modality dominance findings for each emotion category were in line with previous studies. Specifically, Paulmann and Pell (2011) showed face dominance for anger, disgust, and happiness. Collignon et al. (2008) also showed face dominance for disgust. Our data are consistent with these studies in that face dominance was suggested by the observed facilitation effects for anger, disgust, and happiness (see middle column of **Table 2**).

It is important that our results revealed that the modality dominance as reflected in the congruency effect can be classified into two patterns in terms of the facilitation effect and the interference effect. The first pattern is that the modality dominance suggested by the congruency effect occurs by adding up the modality dominances reflected by the facilitation effect and the interference effect. For instance, face dominance for happiness was suggested by both the facilitation effect and the interference effect. Face dominance in the congruency effect was shown for happiness by summing the modality dominances suggested by these effects. Another example is the modality dominance for sadness. The modality dominance for sadness was not suggested by both the facilitation and the interference effect. The modality dominance was not shown for sadness by summing these together. The second pattern was that the modality dominance suggested by the congruency effect did not occur by canceling out the facilitation and interference effects. For instance, for anger, face dominance was suggested by the facilitation effect and voice dominance was suggested by the interference effect. Consequently, the modality dominance in the congruency effect was not shown for anger by canceling out these opposing effects. Thus, the modality dominance for each emotion was elaborated by dividing the congruency effect into the facilitation and interference effects.

### CONCLUSION AND ISSUES FOR FUTURE RESEARCH

In conclusion, our results revealed that instructions to pay attention to one of two modalities modulated the modality dominance for different emotion categories. In particular, the modality dominance for anger changed according to instructions. This finding was provided by comparing the emotion perception with and without instructions directly. It is important to give the instruction about the attention to set the participants’ attitude and control the participants’ understanding towards the task. This paradigm, the immediate cross-modal bias paradigm (Bertelson and de Gelder, 2004), has been widely used in the field of cross-modal perception. However, NI was given in the emotion perception in a natural environment. Therefore, the emotion perception when NI about the attention is given has still to be investigated.

Importantly, emotion congruency between the face and voice also modulated the modality dominance for each emotion category. That is, the emotion expressed in the unattended modality interacted with the emotion perception in a mandatory manner. Regarding the modality dominance, our results show that the modality dominance suggested by the congruency effect can be explained in terms of the facilitation effect and the interference effect. This methodology can provide additional perspective to behavioral and neuroscience study. By focusing on the facilitation and interference effects as well as the congruency effect, future research can examine the separable cognitive mechanisms and neural substrates of facilitation and interference effects.

We analyzed the accuracy on both congruent and incongruent trials when the instruction was given. The congruency effect was calculated by subtracting the accuracy in incongruent trials from that in congruent trials. Although it is possible to calculate the accuracy in congruent trials in NI, the accuracy in incongruent trials could not be calculated because it is impossible to define whether participants’ responses were right or wrong in these trials. Therefore, we could not examine the congruency effect in NI. Instead, we analyzed the face responses and voice responses in incongruent trials when NI was given. In some trials, the reported emotion was neither the face nor voice. Although we eliminated these responses, the proportion of such responses was different among emotion categories. For example, the proportion of such responses for fear (64.8%) was higher than that for other emotions whereas the proportion of such responses for surprise (12.7%) was lower than that for other emotions. This difference might affect results on the face and voice responses particularly for those emotion characterized by a low hit rate (e.g., fear). Therefore, further research is required to examine such responses.

It remains to be investigated whether the emotional intensity, valence, and arousal of emotion expression affects the emotion perception. In this study, we did not manipulate these features to examine its effects on the emotion perception. If emotional intensity or arousal is strong in either modality, then that emotion will be perceived better from that modality. Therefore, it is possible that these features affected the emotion perception and modality dominance differently for each emotion category. Also the differences in the accuracies for each emotion category might have affected the results. Further study is necessary in which the emotional intensity, valence, and arousal of these stimuli are controlled.

It also remains to be investigated whether there are cultural differences with regard to the modality dominance for each emotion category. Regarding the modality dominance, Paulmann and Pell (2011) showed face dominance for fear, though our results demonstrated voice dominance. Other studies may provide a potential answer to this issue. Tanaka et al. (2010) indicated that Japanese individuals are more attuned to voice processing than are Dutch individuals in the multisensory emotion perception. These findings suggest the need to examine cultural differences in the modality dominance for each emotion category.

## Conflict of Interest Statement

The authors declare that the research was conducted in the absence of any commercial or financial relationships that could be construed as a potential conflict of interest.
